# Association between Urolithiasis and History Proton Pump Inhibitor Medication: A Nested Case-Control Study

**DOI:** 10.3390/jcm11195693

**Published:** 2022-09-26

**Authors:** So Young Kim, Dae Myoung Yoo, Woo Jin Bang, Hyo Geun Choi

**Affiliations:** 1 Department of Otorhinolaryngology-Head & Neck Surgery, CHA Bundang Medical Center, CHA University, Seongnam 13496, Korea; 2 Hallym Data Science Laboratory, Hallym University College of Medicine, Anyang 14068, Korea; 3 Department of Urology, Hallym Sacred Heart Hospital, Hallym University College of Medicine, Anyang 14068, Korea; 4 Department of Otorhinolaryngology-Head & Neck Surgery, Hallym University College of Medicine, Anyang 14068, Korea

**Keywords:** urolithiasis, proton pump inhibitors, risk factors, case-control studies, epidemiology

## Abstract

A few retrospective studies have suggested the risk of urolithiasis associated with the use of proton pump inhibitors (PPIs). The current research intended to estimate the risk of urolithiasis according to previous PPI use. A nested case-control study was conducted using the National Health Insurance Service-National Health Screening Cohort in Korea. A total of 28,962 patients with urolithiasis and 115,848 control participants were selected. The previous prescription history of PPI with days of PPI prescription was collected. To calculate the odds ratios (OR) of past, current, and days of PPI use for urolithiasis, logistic regression models were used. Subgroup analyses were conducted. The urolithiasis group demonstrated a higher rate of current PPI users than the control group (60.9% vs. 43.7%). The current PPI users indicated 2.49 times higher odds for urolithiasis than no PPI users (95% confidence intervals [CI] = 2.33–2.66). A longer duration of PPI use was associated with greater odds for urolithiasis (adjusted OR = 1.65 (95% CI = 1.54–1.77) < 1.97 (95% CI = 1.84–2.11) < 2.32 (95% CI = 2.14–2.49) for 1–19 days, 30–364 days, and 365 or more days of PPI prescription). All subgroup analyses described a consistently positive association of previous PPI use with urolithiasis. Prior PPI use was related to a higher risk of urolithiasis. The relationship between previous PPI use and urolithiasis demonstrated a dose-response association.

## 1. Introduction

Proton pump inhibitors (PPIs) are one of the most commonly prescribed medicines worldwide [1]. The prescription of PPIs can be considered when one needs to relieve the symptoms of dyspepsia as well as to prevent gastrointestinal bleeding due to antiplatelet use. PPIs have been widely used with broadened indications for prescription and few concerns about potentially hazardous health effects. However, a growing number of publications have accumulated to warn of the adverse effects of PPIs [1,2]. The listed adverse effects of PPIs have included gastrointestinal tract infection, gastric cancer, dementia, deficiencies in micronutrients, and renal disease [1,2]. Most clinical studies on the adverse effects of PPI have been conducted based on retrospective or cross-sectional study designs, and the causality of PPI use on these adverse effects has been criticized for this lack of temporal association and the requirement for randomized controlled trials [3]. The dose-response associations for PPI use and diseases can enhance the evidence for the adverse effects of PPI use.

Urolithiasis is a prevalent disease with an increasing tendency across the world [4,5]. The prevalence of urolithiasis was estimated to be approximately 12% in North America and 5–9% in Europe [6]. In Korea, approximately 6% of men and 1.8% of women suffer from urolithiasis over a lifetime [7]. The pathogenesis of urolithiasis is complex and involves both environmental and genetic factors [8]. Renal calcium crystallization can be accelerated by oxidative stress and activation of apoptosis initiated by a number of chronic diseases, such as cardiovascular disorders and chronic kidney diseases, and genetic susceptibilities [8]. In addition, the disturbance of magnesium and citrate concentrations can increase calcium oxalate crystallization [9]. Because PPI use was related to chronic diseases and changes in magnesium and citrate levels, these retrospective studies suggested that PPI use can increase the occurrence of urolithiasis [10,11].

We supposed that PPI use could elevate the occurrence of urolithiasis. In addition, the impact of PPI on urolithiasis was presumed to have a dose-response association. To evaluate these assumptions, national health claim data with national hearing screening results were analyzed for the association of the PPI prescription histories with the subsequent occurrence of urolithiasis. To examine the dose-response association between PPI use and the occurrence of urolithiasis, the duration of PPI prescription was calculated, and the relationship with the occurrence of urolithiasis was analyzed according to the duration of PPI use.

## 2. Methods

### 2.1. Study Participants

We used the Korean National Health Insurance Service-Health Screening Cohort data from 2002 to 2015 [12]. The Institutional Review Board (IRB) of Hallym University (IRB No: 2019-10-023) permitted the current research.

**Figure 1:**
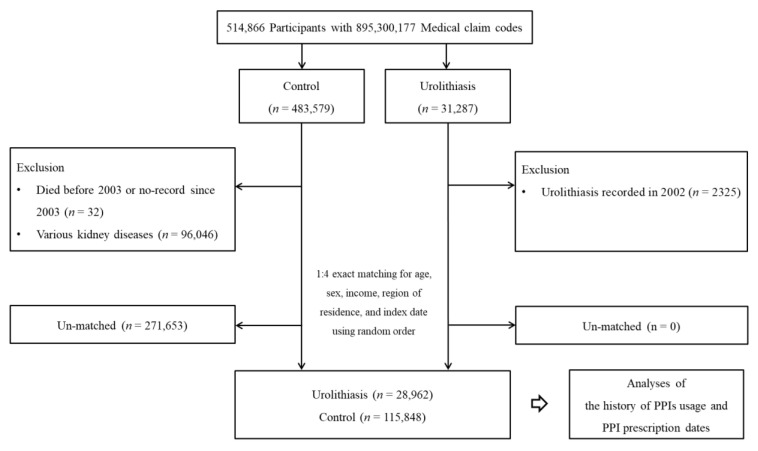
A schematic illustration of the participant selection process used in the present study. Of a total of 514,866 participants, 28,962 urolithiasis participants were matched with 115,848 control participants for age, sex, income, and region of residence.

Among a total of 514,866 with 895,300,177 medical claim codes, participants with urolithiasis were enrolled. Participants diagnosed with urolithiasis in 2002 were excluded. Participants who died before 2003 or had no records since 2003 were excluded. The control participants who were diagnosed with one of the various kidney diseases at least one time (ICD-10 codes: N00-N20, *n* = 96,046) were excluded. The urolithiasis participants and control participants were randomly matched for age, sex, income, and region of residence. At last, 28,962 urolithiasis participants and 115,848 control participants were enrolled (Figure 1).

### 2.2. Medication History

The prescription histories of the proton pump inhibitor (PPI) were collected within a year (365 days) before the diagnosis of urolithiasis. The prescription histories of PPI were categorized according to the presence of the prescription history of PPI and the duration of PPI prescription.

According to the prescription history of PPI, the current PPI users were the participants who were prescribed PPI within 30 days before the diagnosis of urolithiasis. The past PPI users were the participants who were prescribed PPI within 31 days to 365 days before the diagnosis of urolithiasis.

The groups of PPI non-users, PPI prescription dates < 30 days, PPI prescription dates 30 to 364 days, and PPI prescription dates ≥ 1 year (365 days) were classified according to the duration of PPI prescription.

### 2.3. Classification of Diseases and Variables

Urolithiasis (N20) was classified according to ≥2 clinical visits [13].

Age groups were categorized into 10 groups with 5-year intervals [14]. Income groups and regions of residence were classified [14]. Tobacco smoking, alcohol consumption, and obesity using the body mass index (BMI, kg/m^2^) were collected from health check-up data [12].

The total cholesterol (mg/dL), systolic blood pressure (SBP, mmHg), diastolic blood pressure (DBP, mmHg), and fasting blood glucose (mg/dL) were measured.

The Charlson Comorbidity Index (CCI) score was categorized [15].

Gastroesophageal reflux disease (GERD, K21) was classified based on ≥ 2 clinical visits and a history of PPI prescription dates ≥2 weeks.

The prescription histories of H2 blocker and nonsteroidal anti-inflammatory drug (NSAID) prescription were collected within a year before the diagnosis of urolithiasis.

### 2.4. Statistical Analysis

Propensity score (PS) overlap weightings were conducted. PS was estimated using multivariable logistic regression. Urolithiasis participants were weighted by the probability of PS, and control participants were weighted by the probability of 1-PS [16]. The standardized difference (sd) was calculated to compare variables between the urolithiasis and control groups.

The logistic regression was conducted, and the odds ratios (ORs) with 95% confidence intervals (CIs) of current PPI users for urolithiasis and of PPI prescription dates ≥ 365 days for urolithiasis were estimated. The age, sex, income, region of residence, total cholesterol, SBP, DBP, fasting blood glucose, CCI score, prescription dates within 1 year of each H2 blocker and NSAID, and number of GERD treatments within 1 year were adjusted.

Secondary analyses were conducted according to the age, sex, income, region of residence, obesity, smoking status, alcohol consumption, total cholesterol, blood pressure, and fasting blood glucose.

SAS version 9.4 (SAS Institute Inc., Cary, NC, USA) was utilized. The *p* values < 0.05 were set as statistically significant.

## 3. Results

The PPI prescription history was higher in the urolithiasis group than in the control group (sd = 0.36, Table 1). A total of 60.9% of the urolithiasis group and 43.7% of the control group were current PPI users. The PPI prescription dates were longer in the urolithiasis group than in the control group (187.0 [standard deviation (SD) = 222.2] days vs. 148.6 [199.7] days, sd = 0.19). The prescription dates of H2 blockers and NSAIDs and the number of GERD treatments were greater in the urolithiasis group than in the control group. Following overlap weighting adjustment, 60.5% of the urolithiasis group and 45.7% of the control group were current PPI users (sd = 0.31). The PPI prescription dates were 182.8 (SD = 195.1) days for the urolithiasis group and 161.3 (SD = 93.2) days for the control group (sd = 0.14).

Compared to non-PPI users, both past and current PPI users demonstrated higher odds for urolithiasis (both *p* < 0.001, Table 2). The adjusted OR [aOR] for urolithiasis was 1.37 (95% CI = 1.29–1.47) in past PPI users and 2.49 (95% CI = 2.33–2.66) for current PPI users. In addition, longer dates of PPI prescription were related to higher odds for urolithiasis. The adjusted ORs for urolithiasis were 1.65 (95% CI = 1.54–1.77), 1.97 (95% CI = 1.84–2.11), and 2.31 (95% CI = 2.14–2.49) for 1–19 days, 30–364 days, and 365 or more days of PPI prescription, respectively (all *p* < 0.001).

**Figure 2:**
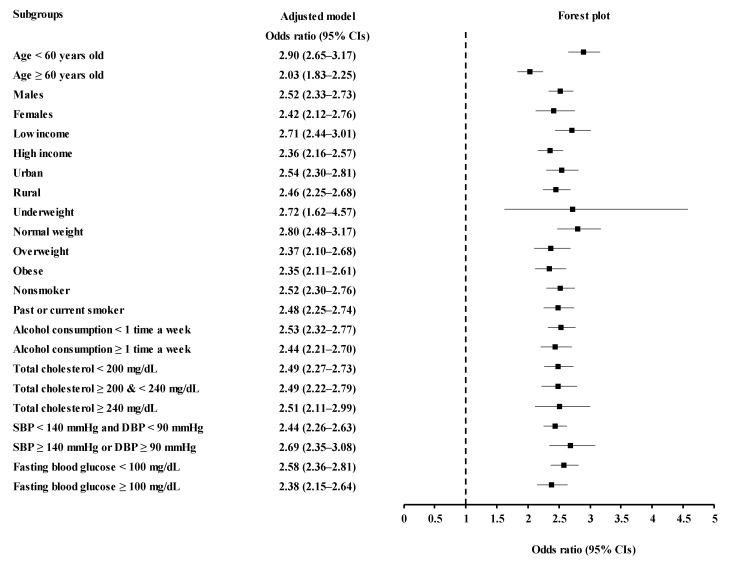
Odds ratios of PPI users for urolithiasis according to age, sex, income, region of residence, obesity, smoking status, alcohol consumption, total cholesterol, blood pressure, and fasting blood glucose.

**Figure 3:**
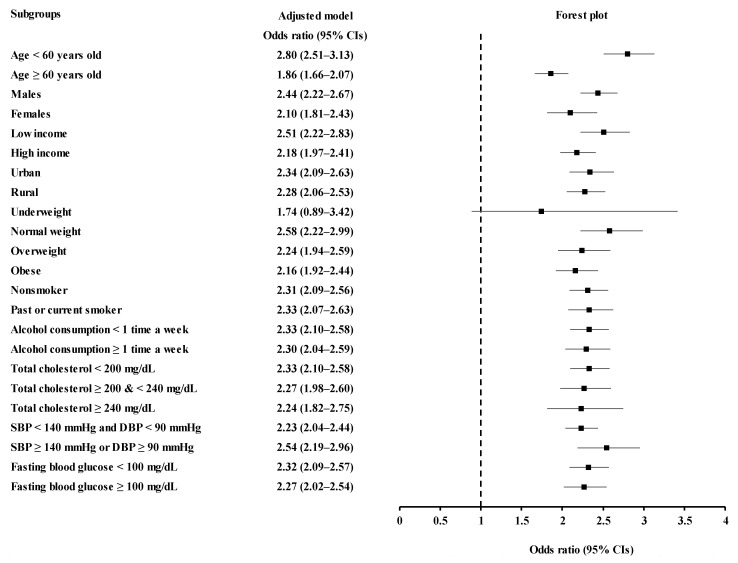
Odds ratios of PPI prescription dates ≥ 365 days for urolithiasis according to age, sex, income, region of residence, obesity, smoking status, alcohol consumption, total cholesterol, blood pressure, and fasting blood glucose.

Subgroup analyses according to age, sex, income, region of residence, obesity, smoking, alcohol consumption, total cholesterol, SBP, and fasting blood glucose indicated a consistent association of PPI use and the date of PPI use with increased odds for urolithiasis (Figure 2 and Figure 3, Supplementary Tables S1 and S2).

## 4. Discussion

Past and current PPI use were related to a higher risk of urolithiasis in the adult population. In addition, a longer duration of PPI use was associated with a greater risk of urolithiasis in this study. This implied a dose-dependent association of PPI use with the risk of urolithiasis. The present data widened previous findings on the impact of PPI use on the occurrence of urolithiasis by adding an analysis of dose-response associations in a large cohort population.

A number of prior studies indicated the adverse impact of PPI use on urolithiasis [10,11]. A retrospective study in patients with GERD demonstrated that PPI use was related to a 1.19-fold higher risk of urolithiasis (95% CI = 1.06–1.34) [10]. Another retrospective study estimated that patients with PPI use had a 1.25-fold higher risk of urolithiasis than those without PPI use (95% CI = 1.19–1.33) [11]. In addition, they found that a higher dose of PPI was associated with a greater risk of urolithiasis over a three-month period (HR = 1.11, 95% CI = 1.09–1.14) [11]. In the current study, a longer duration of PPI use over a one-year period demonstrated a greater risk of urolithiasis than a shorter PPI use. The impacts on urine contents of magnesium and citrate and the susceptibility to infection and chronic diseases can be linked with a higher risk of urolithiasis in patients with PPI use.

Acid suppression by PPI use could increase the risk of urolithiasis. The solubility of calcium oxalate was influenced by magnesium and citrate levels in artificial and natural urine [9]. Magnesium is acknowledged to be a competitive inhibitor of calcium that binds with oxalate [17]. Thus, a decrease in magnesium can increase the risk of calcium oxalate stone. The long-term use of PPIs has been documented to result in hypomagnesemia [18]. A meta-analysis estimated that the pooled adjusted odds ratio of PPI use for hypomagnesemia was 1.71 (95% CI = 1.33–2.19) [18]. In addition, patients with PPI use demonstrated a 12% lower urinary citrate excretion [19]. Citrate inhibits the formation of urolithiasis by binding with calcium and is excreted as a soluble calcium citrate complex [20]. Thus, the decrease of the urinary citrate concentration can elevate the risk of calcium oxalate stone. It was also reported that patients with PPI use had lower urinary citrate and magnesium concentrations in 24-h urine data (both *p* < 0.05) [10]. On the other hand, the urinary oxalate concentration was not significantly different between PPI users and PPI non-users [10,19]. Thus, the lower urinary citrate and magnesium levels in patients with PPI use can mediate an increased risk of urolithiasis.

An increased risk of infection and chronic renal diseases can influence the risk of urolithiasis in PPI users. The suppression of acid in PPI users has been suggested to increase the risk of infection [21]. A number of observational studies have linked the use of acid-suppressing drugs with the infections of various organs, including enteric, skin, respiratory, and systemic infections [21]. Moreover, several observational studies have reported a higher risk of chronic kidney disease in PPI users [22]. PPI users demonstrated a 1.76-fold higher risk of chronic kidney disease than non-PPI users in a multicenter study [22]. Urinary infection and chronic kidney disease are predisposing factors for urolithiasis. In addition, metabolic diseases, such as diabetes and osteoporosis, were reported to be associated with the risk of urolithiasis [23,24]. Because prolonged PPI use is suggested to increase the risk of these metabolic diseases, a greater risk of urolithiasis in PPI users can likely be predicted [25,26].

The association of PPI use with urolithiasis was valid in both short-term and long-term PPI use in this study. Because the formation of a calcium oxalate stone can involve a long period of time stretching over a few years, the impacts of the short-term use of PPI may not be evident within a short follow-up duration. However, the long follow-up duration, from 2002 to 2015, of this study may even result in a significant association of short-term PPI use with the occurrence of urolithiasis. In addition, all subgroups according to demographic and lifestyle factors showed a consistent relationship of PPI use with a greater risk of urolithiasis. Previous studies on the adverse effects of PPI have been criticized due to the older age and higher rates of comorbidities compared to the control population [27]. However, in the present study, the patients with urolithiasis and control participants were matched for age and sex, and overlap propensity score weighting was conducted to minimize potential confounding effects from these factors. As a result, the current data also indicated the possible adverse impact of PPI use on the occurrence of urolithiasis.

This study used a nationwide population cohort. Control participants could be randomly selected without concerns about selection bias. Numerous potential confounders related to demographic, socioeconomic, past medical history, and lifestyle factors were collected and adjusted in the analyses. The prescription histories of PPI were achieved by the health claim data that were prescribed by the physician. In Korea, all Koreans should be registered in the national health insurance program, and the diagnosis and prescription data are legally managed by NHIS. Thus, there was little risk of missing data in this study. However, the severity and management of urolithiasis were heterogeneous in this study. For PPI prescriptions, the types of PPI were not considered in this study. In addition, the compliance of PPI prescriptions could not be checked in this cohort. For urolithiasis, we cannot discriminate the types of urolithiasis. The urinary pH and urinary concentrations of citrate, magnesium, and oxalate were not examined in the present study. At last, due to the retrospective study design, the causality between PPI use and urolithiasis could not be determined in this study. Future studies with selected diagnostic and PPI dosages and a longitudinal study design can solve the current limitations. Although the current findings are limited without randomized controlled trials, clinicians need to consider the potential adverse effects of long-term PPI use on the occurrence of urolithiasis.

## 5. Conclusions

Patients with previous PPI use demonstrated a higher risk of urolithiasis in the adult population. In addition, a longer duration of PPI use was associated with a greater risk of urolithiasis occurrence than a shorter PPI use. Caution is warranted for long-term PPI prescriptions in terms of the risk of urolithiasis.

## Figures and Tables

**Table 1:** General characteristics of participants after overlap propensity score weight.

Characteristics	Before Overlap Weighting Adjustment		Standardized Difference	After Overlap Weighting Adjustment		Standardized Difference
	Urolithiasis	Control		Urolithiasis	Control	
Number	28,962	115,848		22,917	22,917	
Age (years, mean, SD)	58.9 (9.3)	58.9 (9.4)	0.00	58.9 (8.3)	58.9 (4.2)	0.00
Age (years, *n*, %)			0.00			0.01
40–44	1170 (4.0)	4680 (4.0)		926 (4.0)	925 (4.0)	
45–49	3573 (12.3)	14,292 (12.3)		2829 (12.4)	2832 (12.4)	
50–54	5442 (18.8)	21,768 (18.8)		4315 (18.8)	4307 (18.8)	
55–59	6124 (21.1)	24,496 (21.1)		4856 (21.2)	4817 (21.0)	
60–64	4966 (17.2)	19,864 (17.2)		3925 (17.1)	3938 (17.2)	
65–69	3509 (12.1)	14,036 (12.1)		2760 (12.0)	2809 (12.3)	
70–74	2289 (7.9)	9156 (7.9)		1803 (7.9)	1825 (8.0)	
75–79	1288 (4.5)	5152 (4.5)		1022 (4.5)	1012 (4.4)	
80–84	465 (1.6)	1860 (1.6)		370 (1.6)	354 (1.6)	
85+	136 (0.5)	544 (0.5)		109 (0.5)	98 (0.4)	
Sex (*n*, %)			0.00			0.00
Males	18,595 (64.2)	74,380 (64.2)		14,706 (64.2)	14,706 (64.2)	
Females	10,367 (35.8)	41,468 (35.8)		8211 (35.8)	8211 (35.8)	
Income (*n*, %)			0.00			0.00
1 (lowest)	4067 (14.0)	16,268 (14.0)		3214 (14.0)	3227 (14.1)	
2	3380 (11.7)	13,520 (11.7)		2675 (11.7)	2659 (11.6)	
3	4437 (15.3)	17,748 (15.3)		3507 (15.3)	3504 (15.3)	
4	6363 (22.0)	25,452 (22.0)		5034 (22.0)	5036 (22.0)	
5 (highest)	10,715 (37.0)	42,860 (37.0)		8487 (37.0)	8491 (37.1)	
Region of residence (*n*, %)			0.00			0.00
Urban	12,689 (43.8)	50,756 (43.8)		10,047 (43.8)	10,047 (43.8)	
Rural	16,273 (56.2)	65,092 (56.2)		12,870 (56.2)	12,870 (56.2)	
Total cholesterol level (mg/dL, mean, SD)	199.4 (38.4)	198.2 (37.8)	0.03	199.2 (34.1)	199.2 (17.0)	0.00
SBP (mmHg, mean, SD)	126.7 (15.8)	126.4 (16.2)	0.02	126.6 (14.0)	126.6 (7.2)	0.00
DBP (mmHg, mean, SD)	78.8 (10.4)	78.5 (10.6)	0.03	78.8 (9.3)	78.8 (4.7)	0.00
Fasting blood glucose level (mg/dL, mean, SD)	126.7 (15.8)	126.4 (16.2)	0.04	101.6 (25.3)	101.6 (13.5)	0.00
Obesity (*n*, %) ^‡^			0.17			0.00
Underweight	400 (1.4)	2642 (2.3)		346 (1.5)	346 (1.5)	
Normal	8168 (28.2)	40,068 (34.6)		6746 (29.4)	6746 (29.4)	
Overweight	8350 (28.8)	32,404 (28.0)		6594 (28.8)	6594 (28.8)	
Obese I	10,958 (37.8)	37,532 (32.4)		8427 (36.8)	8427 (36.8)	
Obese II	1086 (3.8)	3202 (2.8)		804 (3.5)	804 (3.5)	
Smoking status (*n*, %)			0.06			0.00
Nonsmoker	18,448 (63.7)	71,624 (61.8)		14,517 (63.3)	14,517 (63.3)	
Past smoker	4937 (17.1)	19,156 (16.5)		3889 (17.0)	3889 (17.0)	
Current smoker	5577 (19.3)	25,068 (21.6)		4511 (19.7)	4511 (19.7)	
Alcohol consumption (*n*, %)			0.08			0.00
<1 time a week	18,182 (62.8)	68,020 (58.7)		14,199 (62.0)	14,199 (62.0)	
≥1 time a week	10,780 (37.2)	47,828 (41.3)		8719 (38.0)	8719 (38.0)	
CCI score (score, mean, SD)	1.0 (1.6)	0.8 (1.5)	0.11	0.9 (1.4)	0.9 (0.7)	0.00
CCI score (*n*, %)			0.15			0.08
0 score	17,096 (59.0)	76,694 (66.2)		13,739 (60.0)	14,492 (63.2)	
1 score	5247 (18.1)	17,436 (15.1)		4134 (18.0)	3525 (15.4)	
≥2 scores	6619 (22.9)	21,718 (18.8)		5045 (22.0)	4900 (21.4)	
H2 blocker prescription dates (days, mean, SD)	19.8 (52.0)	15.8 (48.6)	0.08	18.9 (44.4)	18.9 (24.6)	0.00
NSAID prescription dates (days, mean, SD)	24.0 (53.5)	17.9 (46.5)	0.12	22.5 (44.5)	22.5 (25.3)	0.00
No. of GERD treatments (No., mean, SD)	0.5 (1.8)	0.4 (1.7)	0.07	0.5 (1.5)	0.5 (1.1)	0.00
No. of GERD treatments (*n*, %)			0.13			0.09
0 time	24,137 (83.3)	101,724 (87.8)		19,176 (83.7)	19,861 (86.7)	
1 time	1947 (6.7)	5619 (4.9)		1533 (6.7)	1138 (5.0)	
≥2 times	2878 (9.9)	8505 (7.3)		2208 (9.6)	1918 (8.4)	
PPI prescription history (*n*, %)			0.36			0.31
PPI non-user	2153 (7.4)	16,225 (14.0)		1740 (7.6)	2992 (13.1)	
Past PPI user	9166 (31.7)	49,026 (42.3)		7325 (32.0)	9453 (41.3)	
Current PPI user	17,643 (60.9)	50,597 (43.7)		13,853 (60.5)	10,472 (45.7)	
PPI prescription dates (days, mean, SD)	187.0 (222.2)	148.6 (199.7)	0.19	182.8 (195.1)	161.3 (93.2)	0.14
PPI prescription dates (*n*, %)			0.26			0.20
PPI non-user	2156 (7.4)	16,231 (14.0)		1742 (7.6)	2993 (13.1)	
≥1 days & <30 days PPI user	8404 (29.0)	37,569 (32.4)		6760 (29.5)	7094 (31.0)	
≥30 days & <365 days PPI user	11,753 (40.6)	42,442 (36.6)		9287 (40.5)	8546 (37.3)	
≥365 days PPI user	6649 (23.0)	19,606 (16.9)		5128 (22.4)	4285 (18.7)	

Abbreviations: CCI, Charlson Comorbidity Index; DBP, diastolic blood pressure; GERD, gastro-esophageal reflux disease; NSAID, non-steroidal anti-inflammatory drug; PPI, proton pump inhibitor; SBP, systolic blood pressure; SD, standard deviation. ^‡^ Obesity (BMI, body mass index, kg/m^2^) was categorized as <18.5 (underweight), ≥18.5 to <23 (normal), ≥23 to <25 (overweight), ≥25 to <30 (obese I), and ≥30 (obese II).

**Table 2:** Odd ratios (95% confidence interval) of PPI prescription history and PPI prescription dates for urolithiasis.

Characteristics	Urolithiasis	Control	Odds Ratios (95% Confidence Intervals)			
	(Exposure/Total, %)	(Exposure/Total, %)	Crude	*p*-Value	Adjusted Model with OW ^†^	*p*-Value
User of PPI						
Past PPI user	9166/58,192 (15.8)	49,026/58,192 (84.3)	1.33 (1.25–1.42)	<0.001 *	1.37 (1.29–1.47)	<0.001 *
Current PPI user	17,643/68,240 (25.9)	50,597/68,240 (74.2)	2.28 (2.13–2.43)	<0.001 *	2.49 (2.33–2.66)	<0.001 *
PPI dates						
≥1 days & <30 days	8404/45,973 (18.3)	37,569/45,973 (81.7)	1.64 (1.53–1.75)	<0.001 *	1.65 (1.54–1.77)	<0.001 *
≥30 days & <365 days	11,753/54,195 (21.7)	42,442/54,195 (78.3)	1.87 (1.75–1.99)	<0.001 *	1.97 (1.84–2.11)	<0.001 *
≥365 days	6649/26,255 (25.3)	19,606/26,255 (74.7)	2.06 (1.91–2.21)	<0.001 *	2.31 (2.14–2.49)	<0.001 *

Abbreviations: CCI, Charlson comorbidity index; DBP, diastolic blood pressure; GERD, gastro-esophageal reflux disease; NSAID, non-steroidal anti-inflammatory drug; OW, overlap weighting; PPI, proton pump inhibitor; SBP, systolic blood pressure. * Logistic regression model, Significance at *p* < 0.05. ^†^ Adjusted for age, sex, income, region of residence, obesity, smoking status, alcohol consumption, total cholesterol, SBP, DBP, fasting blood glucose, CCI score, NSAID dates, H2 blocker dates, and the number of GERD treatments.

## Data Availability

Releasing the data by the researcher is not legally permitted. All data are available from the database of the Korea Centers for Disease Control and Prevention. The Korea Centers for Disease Control and Prevention allows data access, at a particular cost, for any researcher who promises to follow the research ethics. The data of this article can be downloaded from the website after agreeing to follow the research ethics.

## Supplementary Materials

The following supporting information can be downloaded at: https://www.mdpi.com/article/10.3390/jcm11195693/s1, Table S1: Subgroup analyses regarding odds ratio (95% confidence intervals) of PPI prescription history for urolithiasis according to age, sex, income, region of residence, obesity, smoking state, alcohol consumption, total cholesterol, blood pressure, and fasting blood glucose; Table S2: Subgroup analyses regarding odds ratio (95% confidence intervals) of PPI prescription dates for urolithiasis according to age, sex, income, region of residence, obesity, smoking state, alcohol consumption, total cholesterol, blood pressure, and fasting blood glucose.
