# Supplementary material for: Association between Urolithiasis and History Proton Pump Inhibitor Medication: A Nested Case-Control Study
Source: J Clin Med. 2022 Sep 26;11(19):5693. doi: 10.3390/jcm11195693 (PMC9571377; doi:10.3390/jcm11195693)
Supplement: Supplementary file 1 [file jcm-11-05693-s001.zip › jcm-1920812-supplementary.pdf]

Table S1. Subgroup analyses regarding odds ratio (95% confidence intervals) of PPI prescription history for urolithiasis according to age, sex, income, region of residence, obesity, smoking state, alcohol consumption, total cholesterol, blood pressure, and fasting blood glucose

PPI prescription history	Urolithiasis	Control	Odds ratios (95% confidence intervals)			
	(exposure/total, %)	(exposure/total, %)	Crude	P value	Adjusted model with OW†	P value
Age < 60 years old (n = 81,545)						
Past PPI user	5,721/35,987 (15.9)	30,266/35,987 (84.1)	1.55 (1.45-1.66)	<0.001*	1.52 (1.39-1.66)	<0.001*
Current PPI user	9,393/34,540 (27.2)	25,147/34,540 (72.8)	3.07 (2.88-3.28)	<0.001*	2.90 (2.65-3.17)	<0.001*
Age ≥ 60 years old (n = 63,265)						
Past PPI user	3,445/22,205 (15.5)	18,760/22,205 (84.5)	1.23 (1.14-1.33)	<0.001*	1.19 (1.07-1.32)	0.001*
Current PPI user	8,250/33,700 (24.5)	25,450/33,700 (75.5)	2.17 (2.02-2.33)	<0.001*	2.03 (1.83-2.25)	<0.001*
Males (n = 92,975)						
Past PPI user	6,251/38,408 (16.3)	32,157/38,408 (83.7)	1.45 (1.37-1.53)	<0.001*	1.40 (1.30-1.52)	<0.001*
Current PPI user	10,685/40,552 (26.4)	29,867/40,552 (73.7)	2.66 (2.52-2.82)	<0.001*	2.52 (2.33-2.73)	<0.001*
Females (n = 51,835)						

Past PPI user	2,915/19,784 (14.7)	16,869/19,784 (85.3)	1.35 (1.22-1.50)	<0.001*	1.30 (1.14-1.49)	<0.001*
Current PPI user	6,958/27,688 (25.1)	20,730/27,688 (74.9)	2.63 (2.39-2.90)	<0.001*	2.42 (2.12-2.76)	<0.001*
Low income (n = 59,420)						
Past PPI user	3,600/22,890 (15.7)	19,290/22,890 (84.3)	1.51 (1.40-1.64)	<0.001*	1.48 (1.34-1.65)	<0.001*
Current PPI user	7,428/28,728 (25.9)	21,300/28,728 (74.1)	2.83 (2.62-3.05)	<0.001*	2.71 (2.44-3.01)	<0.001*
High income (n = 85,390)						
Past PPI user	5,566/35,302 (15.8)	29,736/35,302 (84.2)	1.34 (1.26-1.43)	<0.001*	1.31 (1.20-1.42)	<0.001*
Current PPI user	10,215/39,512 (25.9)	29,297/39,512 (74.2)	2.49 (2.34-2.66)	<0.001*	2.36 (2.16-2.57)	<0.001*
Urban (n = 63,445)						
Past PPI user	4,090/25,934 (15.8)	21,844/25,934 (84.2)	1.42 (1.32-1.54)	<0.001*	1.39 (1.26-1.53)	<0.001*
Current PPI user	7,647/29,317 (26.1)	21,670/29,317 (73.9)	2.68 (2.50-2.89)	<0.001*	2.54 (2.30-2.81)	<0.001*
Rural (n = 81,365)						
Past PPI user	5,076/32,258 (15.7)	27,182/32,258 (84.3)	1.40 (1.31-1.49)	<0.001*	1.36 (1.25-1.49)	<0.001*
Current PPI user	9,996/38,923 (25.7)	28,927/38,923 (74.3)	2.59 (2.42-2.76)	<0.001*	2.46 (2.25-2.68)	<0.001*
Underweight (n = 3,042)						
Past PPI user	107/1,181 (9.1)	1,074/1,181 (90.9)	1.26 (0.85-1.88)	0.248	1.16 (0.68-1.96)	0.593

Current PPI user	258/1,382 (18.7)	1,124/1,382 (81.3)	2.91 (2.01-4.21)	<0.001*	2.72 (1.62-4.57)	<0.001*
Normal weight (n = 48,236)						
Past PPI user	2,707/20,158 (13.4)	17,451/20,158 (86.6)	1.56 (1.42-1.71)	<0.001*	1.52 (1.34-1.72)	<0.001*
Current PPI user	4,870/21,547 (22.6)	16,677/21,547 (77.4)	2.94 (2.68-3.21)	<0.001*	2.80 (2.48-3.17)	<0.001*
Overweight (n = 40,754)						
Past PPI user	2,673/16,496 (16.2)	13,823/16,496 (83.8)	1.35 (1.23-1.48)	<0.001*	1.33 (1.17-1.50)	<0.001*
Current PPI user	5,023/19,045 (26.4)	14,022/19,045 (73.6)	2.50 (2.29-2.73)	<0.001*	2.37 (2.10-2.68)	<0.001*
Obese (n = 52,778)						
Past PPI user	3,679/20,357 (18.1)	16,678/20,357 (81.9)	1.34 (1.23-1.45)	<0.001*	1.31 (1.18-1.46)	<0.001*
Current PPI user	7,492/26,266 (28.5)	18,774/26,266 (71.5)	2.41 (2.24-2.61)	<0.001*	2.35 (2.11-2.61)	<0.001*
Nonsmoker (n = 90,072)						
Past PPI user	5,604/35,453 (15.8)	29,849/35,453 (84.2)	1.42 (1.33-1.52)	<0.001*	1.39 (1.27-1.53)	<0.001*
Current PPI user	11,756/45,294 (26.0)	33,538/45,294 (74.1)	2.65 (2.48-2.84)	<0.001*	2.52 (2.30-2.76)	<0.001*
Past or current smoker (n = 54,738)						
Past PPI user	3,562/22,739 (15.7)	19,177/22,739 (84.3)	1.39 (1.30-1.50)	<0.001*	1.36 (1.23-1.50)	<0.001*
Current PPI user	5,887/22,946 (25.7)	17,059/22,946 (74.3)	2.59 (2.41-2.78)	<0.001*	2.48 (2.25-2.74)	<0.001*

Alcohol consumption < 1 time a week (n = 86,202)						
Past PPI user	5,500/33,307 (16.5)	27,807/33,307 (83.5)	1.42 (1.33-1.52)	<0.001*	1.40 (1.28-1.53)	<0.001*
Current PPI user	11,504/43,272 (26.6)	31,768/43,272 (73.4)	2.60 (2.43-2.77)	<0.001*	2.53 (2.32-2.77)	<0.001*
Alcohol consumption ≥ 1 time a week (n = 58,608)						
Past PPI user	3,666/24,885 (14.7)	21,219/24,885 (85.3)	1.38 (1.28-1.49)	<0.001*	1.34 (1.21-1.48)	<0.001*
Current PPI user	6,139/24,968 (24.6)	18,829/24,968 (75.4)	2.60 (2.42-2.80)	<0.001*	2.44 (2.21-2.70)	<0.001*
Total cholesterol < 200 mg/dL (n = 77,607)						
Past PPI user	4,757/31,477 (15.1)	26,720/31,477 (84.9)	1.38 (1.28-1.47)	<0.001*	1.34 (1.22-1.47)	<0.001*
Current PPI user	9,282/36,402 (25.5)	27,120/36,402 (74.5)	2.64 (2.47-2.83)	<0.001*	2.49 (2.27-2.73)	<0.001*
Total cholesterol ≥ 200 & < 240 mg/dL (n = 47,901)						
Past PPI user	3,123/19,271 (16.2)	16,148/19,271 (83.8)	1.45 (1.33-1.58)	<0.001*	1.41 (1.25-1.58)	<0.001*
Current PPI user	5,860/22,539 (26.0)	16,679/22,539 (74.0)	2.64 (2.43-2.87)	<0.001*	2.49 (2.22-2.79)	<0.001*
Total cholesterol ≥ 240 mg/dL (n = 19,302)						

Past PPI user	1,286/7,444 (17.3)	6,158/7,444 (82.7)	1.45 (1.27-1.65)	<0.001*	1.43 (1.20-1.70)	<0.001*
Current PPI user	2,501/9,299 (26.9)	6,798/9,299 (73.1)	2.56 (2.25-2.90)	<0.001*	2.51 (2.11-2.99)	<0.001*
SBP < 140 mmHg and DBP < 90 mmHg (n = 108,522)						
Past PPI user	7,111/45,239 (15.7)	38,128/45,239 (84.3)	1.37 (1.30-1.45)	<0.001*	1.34 (1.24-1.45)	<0.001*
Current PPI user	12,822/49,404 (26.0)	36,582/49,404 (74.1)	2.58 (2.44-2.73)	<0.001*	2.44 (2.26-2.63)	<0.001*
SBP ≥ 140 mmHg or DBP ≥ 90 mmHg (n = 36,288)						
Past PPI user	2,055/12,953 (15.9)	10,898/12,953 (84.1)	1.53 (1.38-1.70)	<0.001*	1.48 (1.29-1.70)	<0.001*
Current PPI user	4,821/18,836 (25.6)	14,015/18,836 (74.4)	2.80 (2.53-3.09)	<0.001*	2.69 (2.35-3.08)	<0.001*
Fasting blood glucose < 100 mg/dL (n = 88,838)						
Past PPI user	5,640/37,270 (15.1)	31,630/37,270 (84.9)	1.41 (1.32-1.50)	<0.001*	1.38 (1.27-1.51)	<0.001*
Current PPI user	10,307/40,369 (25.5)	30,062/40,369 (74.5)	2.71 (2.54-2.88)	<0.001*	2.58 (2.36-2.81)	<0.001*
Fasting blood glucose ≥ 100 mg/dL (n = 55,972)						
Past PPI user	3,526/20,922 (16.9)	17,396/20,922 (83.2)	1.43 (1.32-1.54)	<0.001*	1.38 (1.24-1.53)	<0.001*
Current PPI user	7,336/27,871 (26.3)	20,535/27,871 (73.7)	2.51 (2.33-2.71)	<0.001*	2.38 (2.15-2.64)	<0.001*

Abbreviations: CCI, Charlson comorbidity index; DBP, diastolic blood pressure; GERD, gastro-esophageal reflux disease; NSAID, non-steroidal anti-inflammatory drug; OW, overlap weighting; PPI, proton pump inhibitor; SBP, systolic blood pressure

* Logistic regression model, Significance at P < 0.05

† Adjusted for age, sex, income, region of residence, obesity, smoking status, alcohol consumption, total cholesterol, SBP, DBP, fasting blood glucose, CCI score, NSAID dates, H2 blocker dates, and the number of GERD treatment

Table S2. Subgroup analyses regarding odds ratio (95% confidence intervals) of PPI prescription dates for urolithiasis according to age, sex, income, region of residence, obesity, smoking state, alcohol consumption, total cholesterol, blood pressure, and fasting blood glucose

PPI prescription dates	Urolithiasis	Control	Odds ratios (95% confidence intervals)			
	(exposure/total, %)	(exposure/total, %)	Crude	P value	Adjusted model with OW†	P value
Age < 60 years old (n = 81,545)						
≥1 days & <30 days	5,393/29,930 (18.0)	24,537/29,930 (82.0)	1.81 (1.69-1.93)	<0.001*	1.78 (1.63-1.95)	<0.001*
≥30 days & < 365 days	7,191/31,372 (22.9)	24,181/31,372 (77.1)	2.45 (2.29-2.61)	<0.001*	2.30 (2.11-2.52)	<0.001*
≥ 365 days	2,530/9,223 (27.4)	6,693/9,223 (72.6)	3.11 (2.88-3.35)	<0.001*	2.80 (2.51-3.13)	<0.001*
Age ≥ 60 years old (n = 63,265)						
≥1 days & <30 days	3,011/16,043 (18.8)	13,032/16,043 (81.2)	1.54 (1.42-1.67)	<0.001*	1.51 (1.36-1.68)	<0.001*
≥30 days & < 365 days	4,562/22,823 (20.0)	18,261/22,823 (80.0)	1.67 (1.55-1.80)	<0.001*	1.57 (1.41-1.74)	<0.001*
≥ 365 days	4,119/17,032 (24.2)	12,913/17,032 (75.8)	2.13 (1.97-2.30)	<0.001*	1.86 (1.66-2.07)	<0.001*
Males (n = 92,975)						
≥1 days & <30 days	5,773/30,813 (18.7)	25,040/30,813 (81.3)	1.72 (1.62-1.82)	<0.001*	1.67 (1.54-1.81)	<0.001*
≥30 days & < 365 days	7,123/32,821 (21.7)	25,698/32,821 (78.3)	2.06 (1.95-2.18)	<0.001*	1.95 (1.80-2.11)	<0.001*

≥ 365 days	4,037/15,319 (26.4)	11,282/15,319 (73.7)	2.66 (2.50-2.83)	<0.001*	2.44 (2.22-2.67)	<0.001*
Females (n = 51,835)						
≥1 days & <30 days	2,631/15,160 (17.4)	12,529/15,160 (82.7)	1.65 (1.49-1.82)	<0.001*	1.61 (1.40-1.84)	<0.001*
≥30 days & < 365 days	4,630/21,374 (21.7)	16,744/21,374 (78.3)	2.17 (1.96-2.39)	<0.001*	1.99 (1.74-2.28)	<0.001*
≥ 365 days	2,612/10,936 (23.9)	8,324/10,936 (76.1)	2.46 (2.22-2.73)	<0.001*	2.10 (1.81-2.43)	<0.001*
Low income (n = 59,420)						
≥1 days & <30 days	2,631/15,160 (17.4)	12,529/15,160 (82.7)	1.82 (1.68-1.98)	<0.001*	1.79 (1.61-2.00)	<0.001*
≥30 days & < 365 days	4,630/21,374 (21.7)	16,744/21,374 (78.3)	2.26 (2.09-2.45)	<0.001*	2.15 (1.94-2.39)	<0.001*
≥ 365 days	2,612/10,936 (23.9)	8,324/10,936 (76.1)	2.76 (2.54-3.00)	<0.001*	2.51 (2.22-2.83)	<0.001*
High income (n = 85,390)						
≥1 days & <30 days	5,064/27,798 (18.2)	22,734/27,798 (81.8)	1.59 (1.49-1.70)	<0.001*	1.56 (1.43-1.71)	<0.001*
≥30 days & < 365 days	6,798/31,502 (21.6)	24,704/31,502 (78.4)	1.97 (1.85-2.10)	<0.001*	1.85 (1.70-2.02)	<0.001*
≥ 365 days	3,918/15,509 (25.3)	11,591/15,509 (74.7)	2.42 (2.26-2.59)	<0.001*	2.18 (1.97-2.41)	<0.001*
Urban (n = 63,445)						
≥1 days & <30 days	3,725/20,405 (18.3)	16,680/20,405 (81.7)	1.70 (1.58-1.83)	<0.001*	1.67 (1.51-1.85)	<0.001*
≥30 days & < 365 days	5,119/23,437 (21.8)	18,318/23,437 (78.2)	2.13 (1.97-2.29)	<0.001*	2.01 (1.81-2.22)	<0.001*

≥ 365 days	2,893/11,406 (25.4)	8,513/11,406 (74.6)	2.59 (2.39-2.80)	<0.001*	2.34 (2.09-2.63)	<0.001*
Rural (n = 81,365)						
≥1 days & <30 days	4,679/25,568 (18.3)	20,889/25,568 (81.7)	1.67 (1.56-1.79)	<0.001*	1.64 (1.50-1.80)	<0.001*
≥30 days & < 365 days	6,634/30,758 (21.6)	24,124/30,758 (78.4)	2.05 (1.92-2.19)	<0.001*	1.94 (1.77-2.13)	<0.001*
≥ 365 days	3,756/14,849 (25.3)	11,093/14,849 (74.7)	2.53 (2.36-2.71)	<0.001*	2.28 (2.06-2.53)	<0.001*
Underweight (n = 3,042)						
≥1 days & <30 days	129/1,020 (12.7)	891/1,020 (87.4)	1.84 (1.24-2.71)	0.002	1.66 (0.98-2.80)	0.059
≥30 days & < 365 days	178/1,140 (15.6)	962/1,140 (84.4)	2.35 (1.61-3.43)	<0.001*	2.07 (1.22-3.51)	0.007
≥ 365 days	58/403 (14.4)	345/403 (85.6)	2.13 (1.37-3.32)	0.001	1.74 (0.89-3.42)	0.106
Normal weight (n = 48,236)						
≥1 days & <30 days	2,569/16,499 (15.6)	13,930/16,499 (84.4)	1.85 (1.68-2.03)	<0.001*	1.80 (1.59-2.04)	<0.001*
≥30 days & < 365 days	3,446/18,029 (19.1)	14,583/18,029 (80.9)	2.37 (2.16-2.60)	<0.001*	2.23 (1.97-2.53)	<0.001*
≥ 365 days	1,560/7,173 (21.8)	5,613/7,173 (78.3)	2.79 (2.52-3.08)	<0.001*	2.58 (2.22-2.99)	<0.001*
Overweight (n = 40,754)						
≥1 days & <30 days	2,422/13,166 (18.4)	10,744/13,166 (81.6)	1.57 (1.43-1.72)	<0.001*	1.55 (1.37-1.75)	<0.001*
≥30 days & < 365 days	3,397/15,134 (22.5)	11,737/15,134 (77.6)	2.02 (1.84-2.21)	<0.001*	1.92 (1.69-2.17)	<0.001*

≥ 365 days	1,876/7,239 (25.9)	5,363/7,239 (74.1)	2.44 (2.21-2.68)	<0.001*	2.24 (1.94-2.59)	<0.001*
Obese (n = 52,778)						
≥1 days & <30 days	3,284/15,288 (21.5)	12,004/15,288 (78.5)	1.66 (1.53-1.80)	<0.001*	1.62 (1.45-1.81)	<0.001*
≥30 days & < 365 days	4,732/19,892 (23.8)	15,160/19,892 (76.2)	1.89 (1.75-2.04)	<0.001*	1.82 (1.63-2.02)	<0.001*
≥ 365 days	3,155/11,440 (27.6)	8,285/11,440 (72.4)	2.31 (2.12-2.50)	<0.001*	2.16 (1.92-2.44)	<0.001*
Nonsmoker (n = 90,072)						
≥1 days & <30 days	5,001/27,522 (18.2)	22,521/27,522 (81.8)	1.68 (1.57-1.80)	<0.001*	1.66 (1.51-1.82)	<0.001*
≥30 days & < 365 days	7,855/35,450 (22.2)	27,595/35,450 (77.8)	2.15 (2.01-2.30)	<0.001*	2.04 (1.86-2.24)	<0.001*
≥ 365 days	4,502/17,771 (25.3)	13,269/17,771 (74.7)	2.57 (2.39-2.76)	<0.001*	2.31 (2.09-2.56)	<0.001*
Past or current smoker (n = 54,738)						
≥1 days & <30 days	3,403/18,451 (18.4)	15,048/18,451 (81.6)	1.70 (1.58-1.83)	<0.001*	1.66 (1.50-1.83)	<0.001*
≥30 days & < 365 days	3,898/18,745 (20.8)	14,847/18,745 (79.2)	1.97 (1.83-2.12)	<0.001*	1.87 (1.69-2.07)	<0.001*
≥ 365 days	2,147/8,484 (25.3)	6,337/8,484 (74.7)	2.54 (2.34-2.75)	<0.001*	2.33 (2.07-2.63)	<0.001*
Alcohol consumption < 1 time a week (n = 86,202)						
≥1 days & <30 days	5,110/26,768 (19.1)	21,658/26,768 (80.9)	1.69 (1.58-1.81)	<0.001*	1.68 (1.53-1.84)	<0.001*

≥30 days & < 365 days	7,620/33,242 (22.9)	25,622/33,242 (77.1)	2.13 (1.99-2.28)	<0.001*	2.06 (1.88-2.25)	<0.001*
≥ 365 days	4,273/16,566 (25.8)	12,293/16,566 (74.2)	2.49 (2.32-2.67)	<0.001*	2.33 (2.10-2.58)	<0.001*
Alcohol consumption ≥ 1 time a week (n = 58,608)						
≥1 days & <30 days	3,294/19,205 (17.2)	15,911/19,205 (82.9)	1.65 (1.53-1.78)	<0.001*	1.62 (1.47-1.80)	<0.001*
≥30 days & < 365 days	4,133/20,953 (19.7)	16,820/20,953 (80.3)	1.96 (1.82-2.11)	<0.001*	1.84 (1.66-2.04)	<0.001*
≥ 365 days	2,376/9,689 (24.5)	7,313/9,689 (75.5)	2.59 (2.39-2.81)	<0.001*	2.30 (2.04-2.59)	<0.001*
Total cholesterol < 200 mg/dL (n = 77,607)						
≥1 days & <30 days	4,394/24,872 (17.7)	20,478/24,872 (82.3)	1.66 (1.54-1.78)	<0.001*	1.63 (1.48-1.79)	<0.001*
≥30 days & < 365 days	5,797/27,874 (20.8)	22,077/27,874 (79.2)	2.03 (1.89-2.17)	<0.001*	1.91 (1.74-2.10)	<0.001*
≥ 365 days	3,846/15,129 (25.4)	11,283/15,129 (74.6)	2.63 (2.45-2.83)	<0.001*	2.33 (2.10-2.58)	<0.001*
Total cholesterol ≥ 200 & < 240 mg/dL (n = 47,901)						
≥1 days & <30 days	2,864/15,430 (18.6)	12,566/15,430 (81.4)	1.71 (1.57-1.87)	<0.001*	1.66 (1.48-1.87)	<0.001*
≥30 days & < 365 days	4,139/18,498 (22.4)	14,359/18,498 (77.6)	2.16 (1.99-2.36)	<0.001*	2.03 (1.80-2.28)	<0.001*
≥ 365 days	1,979/7,877 (25.1)	5,898/7,877 (74.9)	2.52 (2.29-2.76)	<0.001*	2.27 (1.98-2.60)	<0.001*

Total cholesterol ≥ 240 mg/dL (n = 19,302)						
≥1 days & <30 days	1,146/5,671 (20.2)	4,525/5,671 (79.8)	1.76 (1.54-2.01)	<0.001*	1.72 (1.44-2.06)	<0.001*
≥30 days & < 365 days	1,817/7,823 (23.2)	6,006/7,823 (76.8)	2.10 (1.85-2.39)	<0.001*	2.02 (1.70-2.42)	<0.001*
≥ 365 days	824/3,249 (25.4)	2,425/3,249 (74.6)	2.36 (2.05-2.72)	<0.001*	2.24 (1.82-2.75)	<0.001*
SBP < 140 mmHg and DBP < 90 mmHg (n = 108,522)						
≥1 days & <30 days	6,601/36,493 (18.1)	29,892/36,493 (81.9)	1.62 (1.53-1.72)	<0.001*	1.60 (1.48-1.73)	<0.001*
≥30 days & < 365 days	8,727/40,062 (21.8)	31,335/40,062 (78.2)	2.05 (1.94-2.17)	<0.001*	1.93 (1.79-2.09)	<0.001*
≥ 365 days	4,602/18,081 (25.5)	13,479/18,081 (74.6)	2.51 (2.36-2.67)	<0.001*	2.23 (2.04-2.44)	<0.001*
SBP ≥ 140 mmHg or DBP ≥ 90 mmHg (n = 36,288)						
≥1 days & <30 days	1,803/9,480 (19.0)	7,677/9,480 (81.0)	1.91 (1.72-2.12)	<0.001*	1.85 (1.60-2.13)	<0.001*
≥30 days & < 365 days	3,026/14,133 (21.4)	11,107/14,133 (78.6)	2.22 (2.00-2.45)	<0.001*	2.11 (1.84-2.42)	<0.001*
≥ 365 days	2,047/8,174 (25.0)	6,127/8,174 (75.0)	2.72 (2.44-3.02)	<0.001*	2.54 (2.19-2.96)	<0.001*
Fasting blood glucose < 100 mg/dL (n = 88,838)						
≥1 days & <30 days	5,259/30,247 (17.4)	24,988/30,247 (82.6)	1.66 (1.56-1.78)	<0.001*	1.64 (1.50-1.79)	<0.001*

≥30 days & < 365 days	7,361/33,863 (21.7)	26,502/33,863 (78.3)	2.19 (2.06-2.34)	<0.001*	2.08 (1.90-2.27)	<0.001*
≥ 365 days	3,327/13,527 (24.6)	10,200/13,527 (75.4)	2.58 (2.40-2.76)	<0.001*	2.32 (2.09-2.57)	<0.001*
Fasting blood glucose ≥ 100 mg/dL (n = 55,972)						
≥1 days & <30 days	3,145/15,726 (20.0)	12,581/15,726 (80.0)	1.75 (1.62-1.90)	<0.001*	1.70 (1.52-1.89)	<0.001*
≥30 days & < 365 days	4,392/20,332 (21.6)	15,940/20,332 (78.4)	1.93 (1.79-2.09)	<0.001*	1.82 (1.64-2.02)	<0.001*
≥ 365 days	3,322/12,728 (26.1)	9,406/12,728 (73.9)	2.48 (2.29-2.68)	<0.001*	2.27 (2.02-2.54)	<0.001*

Abbreviations: CCI, Charlson comorbidity index; DBP, diastolic blood pressure; GERD, gastro-esophageal reflux disease; NSAID, non-steroidal anti-inflammatory drug; OW, overlap weighting; PPI, proton pump inhibitor; SBP, systolic blood pressure

* Logistic regression model, Significance at P < 0.05

† Adjusted for age, sex, income, region of residence, obesity, smoking status, alcohol consumption, total cholesterol, SBP, DBP, fasting blood glucose, CCI score, NSAID dates, H2 blocker dates, and the number of GERD treatment
